# Concomitant Cholinesterase Inhibitor and Anticholinergic Drug Use Among Older Adults With Dementia

**DOI:** 10.1093/geroni/igaa057.842

**Published:** 2020-12-16

**Authors:** Elizabeth Carter, Erwin Tan

**Affiliations:** AARP, Washington, District of Columbia, United States

## Abstract

Anticholinergic medications (ACh) are frequently prescribed to older adults despite being associated with impaired physical functioning. Moreover, the concomitant use of ACh and cholinesterase inhibitors (ChEI) reduces the body’s response to both drugs, thereby diminishing the modest effectiveness of ChEI at slowing the progression of dementia symptoms. The objective of this study was to assess the risk of concomitant ACH/ChEI use on functional outcomes, including fall, fracture, and traumatic brain injury (TBI). We conducted a retrospective review of data from the OptumLabs® Data Warehouse, a de-identified administrative claims database for commercially insured and Medicare Advantage (MA) enrollees, representing a diverse mixture of ages, ethnicities, and geographical regions across the U.S. Our cohort included adults 65 and older enrolled in MA between 1998 and 2017. Subjects were required to have dementia (by a diagnosis and/or prescription for a dementia drug (memantine or ChEI)) and at least one claim for ChEI during 12 months of follow-up. Subjects had to be enrolled in MA 6 months prior to the dementia index date. We defined concomitant ACh/ChEI use as an overlap of 30 days or more. Nearly one-third (29%) were concomitantly prescribed ACh and ChEI. Half (51%) of concomitant users were prescribed ChEI first, 46% were prescribed ACh first, and 3% received prescriptions on the same day. Results from multiple logistic regression analyses show that older adults with dementia who had concomitant ACh/ChEI use were 18%, 16%, and 25% more likely to experience a fall, fracture, or TBI, respectively, than those taking ChEI alone.

